# The Development of Computational Biology in South Africa: Successes Achieved and Lessons Learnt

**DOI:** 10.1371/journal.pcbi.1004395

**Published:** 2016-02-04

**Authors:** Nicola J. Mulder, Alan Christoffels, Tulio de Oliveira, Junaid Gamieldien, Scott Hazelhurst, Fourie Joubert, Judit Kumuthini, Ché S. Pillay, Jacky L. Snoep, Özlem Tastan Bishop, Nicki Tiffin

**Affiliations:** 1 Computational Biology Division, Department of Integrative Biomedical Sciences, Institute of Infectious Disease and Molecular Medicine, Faculty of Health Sciences, University of Cape Town, Cape Town, South Africa; 2 South African National Bioinformatics Institute/Medical Research Council of South Africa Bioinformatics Unit, University of the Western Cape, Bellville, South Africa; 3 Africa Centre for Health and Population Studies, School of Laboratory Medicine and Medical Sciences, University of KwaZulu-Natal, Durban, South Africa; 4 School of Electrical & Information Engineering, and Sydney Brenner Institute for Molecular Bioscience, University of the Witwatersrand, Johannesburg, South Africa; 5 Centre for Bioinformatics and Computational Biology, University of Pretoria, Pretoria, South Africa; 6 Centre for Proteomic and Genomic Research, Cape Town, South Africa; 7 School of Life Sciences, University of KwaZulu-Natal, Pietermaritzburg, South Africa; 8 Department of Biochemistry, Stellenbosch University, Stellenbosch, South Africa; 9 Research Unit in Bioinformatics (RUBi), Department of Biochemistry and Microbiology, Rhodes University, Grahamstown, South Africa; Hellas, GREECE

## Abstract

Bioinformatics is now a critical skill in many research and commercial environments as biological data are increasing in both size and complexity. South African researchers recognized this need in the mid-1990s and responded by working with the government as well as international bodies to develop initiatives to build bioinformatics capacity in the country. Significant injections of support from these bodies provided a springboard for the establishment of computational biology units at multiple universities throughout the country, which took on teaching, basic research and support roles. Several challenges were encountered, for example with unreliability of funding, lack of skills, and lack of infrastructure. However, the bioinformatics community worked together to overcome these, and South Africa is now arguably the leading country in bioinformatics on the African continent. Here we discuss how the discipline developed in the country, highlighting the challenges, successes, and lessons learnt.

## Introduction

The fields of bioinformatics and computational biology have grown in importance as drivers of research in the life sciences as evidenced by the increasing number of journals and international conferences dedicated to these fields. The need for skills in these areas has grown accordingly in South Africa, as researchers are more frequently awarded grants for projects generating large-scale biological data. Local scientists are generating large and varied datasets including next-generation sequencing (NGS; genomic, transcriptomic, and metagenomic), proteomic data, and other data, coupled with rich phenotypic datasets, especially large patient and surveillance cohorts. These research groups seldom have embedded data analysts, so they turn to bioinformatics groups for support. Fortunately, there are well-established bioinformatics and computational biology groups at many of the universities in South Africa, reflecting the significant resources committed to bioinformatics development in the country since the 1990s. In this article, we outline the growth of bioinformatics in South Africa, highlighting the challenges as well as the impact of government support on the development of the field. Where relevant, we also describe lessons learnt.

## Origins: History and Development of Bioinformatics in South Africa

South Africa is a middle-income country (the World Bank’s 2013 estimate of GDP [Gross Domestic Product] per capita at purchasing power parity was US$12,530), with significant social inequality. While there are resources to develop new scientific fields, demands on government to meet immediate social needs makes neither organic growth nor strategic investment a given. Following the Second World War, state policy and government funding built a strong South African tradition of scientific research. Despite areas of great strength and international leadership, however, there were many neglected areas, policy challenges, and a fragmented science and innovation landscape across both the public and private sector [1–3]. The ending of sanctions and the accession of South Africa to the World Trade Organization reduced protection and state subsidy to some areas of research that had previously been of strategic importance (e.g., some areas of military research) [3].

Bioinformatics development post-1994 fell within a research and development (R&D) policy adopted by the new democratic government to address the legacies of apartheid and economic needs. New science and research policies aimed to develop a coherent approach to building research infrastructure and innovation. Blue-skies research was valued particularly in areas of competitive advantage such as palaeoanthropology, astronomy, and Antarctic studies. A key part of the new research policy including the national biotechnology strategy, however, explicitly aimed to jump-start the economy and address the serious economic challenges left by the apartheid. An ongoing challenge is to address skewed demographics of researchers and the higher education sector that arose from racial inequalities of the apartheid, in which the black majority was largely excluded from science [3]. This apartheid legacy is still apparent two generations later in the failure of the school system to generate enough school completers for the science-based careers that must be part of the growing economy. This is particularly true in areas like bioinformatics, where a strong mathematics training is required [3,4].

### Early phases

The development of bioinformatics in South Africa and on the African continent can be traced back to 1996 when Winston Hide founded the South African National Bioinformatics Institute (SANBI) on the University of the Western Cape (UWC) campus. South Africa’s National Research Foundation (NRF) provided the initial funding to establish SANBI, and this was supplemented with funding from what was then Glaxo Wellcome and the United States Department of Energy. The following two years saw investment from the South African telecommunications company, Telkom. During this time, the first four bioinformatics PhD students were enrolled at SANBI, and by 2000, South Africa’s vision for expanding bioinformatics was realized with the establishment of the South African Medical Research Council Bioinformatics Capacity Development Unit at SANBI with committed funding for 2000–2015. In 2003, a regional bioinformatics training center under the umbrella of the World Health Organization’s Tropical Disease Research (TDR) Program was established at SANBI. This program provided a bioinformatics training mechanism for African researchers–a program that subsequently informed training within the International Glossina Genome Initiative [5].

Government support for biotechnology and bioinformatics was formalized in the 2001 Green Paper on the Biotechnology Strategy [6], which highlighted the importance of human capacity development and designated bioinformatics eligible for critical skills work permit applications. Strategic and practical support continued, with ongoing updates of state policies (e.g., [7,8]). Several universities established nascent bioinformatics groups and lobbied collectively, resulting in an influential call to action published in the *South African Medical Journal* [9].

### The National Bioinformatics Network

The first formal call for establishing a nationwide bioinformatics infrastructure came from the South African Medical Research Council in 2002, with funding from the national Department of Arts, Culture, Science, and Technology and later the Department of Science and Technology (DST). While the amount committed was modest, this catalyzed meetings of the bioinformatics stakeholders in South Africa and subsequent formation of a group of scientists promoting the interests of bioinformatics nationally. Shortly thereafter, the DST announced an initiative to establish Biotechnology Regional Innovation Centers (BRICs), including a semiautonomous National Bioinformatics Network (NBN). Planning for the NBN took place during 2002, and the NBN was given a mandate to generate bioinformatics capability and infrastructure and act as a service provider, with specific emphasis on its role in supporting the BRICs. Calls for proposals to set up nodes for the network and for research projects were received in 2003, and a Director was appointed, followed by administrative, technical and scientific staff. Initially, five nodes were funded at Universities across South Africa, with two further nodes added at a later stage. Node funding covered staff appointments, equipment purchases, general running costs, student bursaries, training courses, and research projects. This was followed by five further annual rounds of substantial funding. The NBN funding ended in 2008, and the NBN as an entity was closed in 2009. The NBN initiative vastly expanded bioinformatics capacity in South Africa through the funding of staff positions and through the training of postgraduate students, as well as through extensively taught short courses for researchers. These activities all fell outside of traditional university commitments. The investment in infrastructure provided a very significant boost to the scope of projects that could be executed and the level of bioinformatics research ongoing.

In retrospect, some of the lessons learned from the NBN initiative may be useful for others. These include:

- Splitting of funds into noncompetitive funding for training and initial infrastructure and competitive funding for research-fostered cooperation between bioinformatics groups, while encouraging healthy competition for funding of research projects.
- The use of an independent, international Scientific Advisory Board (SAB) to mediate key funding decisions ensured quality and nonpolitical funding decisions, particularly at the early phases when the South African bioinformatics community was too small to support independent review at a national level.
- Although the state identified bioinformatics as key to supporting health priorities within the biotech industry, the need to build general infrastructure and research capacity was also recognized, and the NBN was given wide autonomy for the type of research funded.
- A very complex governance structure included the NBN Trust Committee, the SAB, and an executive, and this led to a breakdown in accountability when problems did occur.
- Whilst the dedicated funding was very beneficial, the process was poorly integrated into the national funding system and researchers had to source funding from different entities to cover different aspects of a single project. For example, funding for related laboratory work had to be sourced under different initiatives.

**Fig 1:**
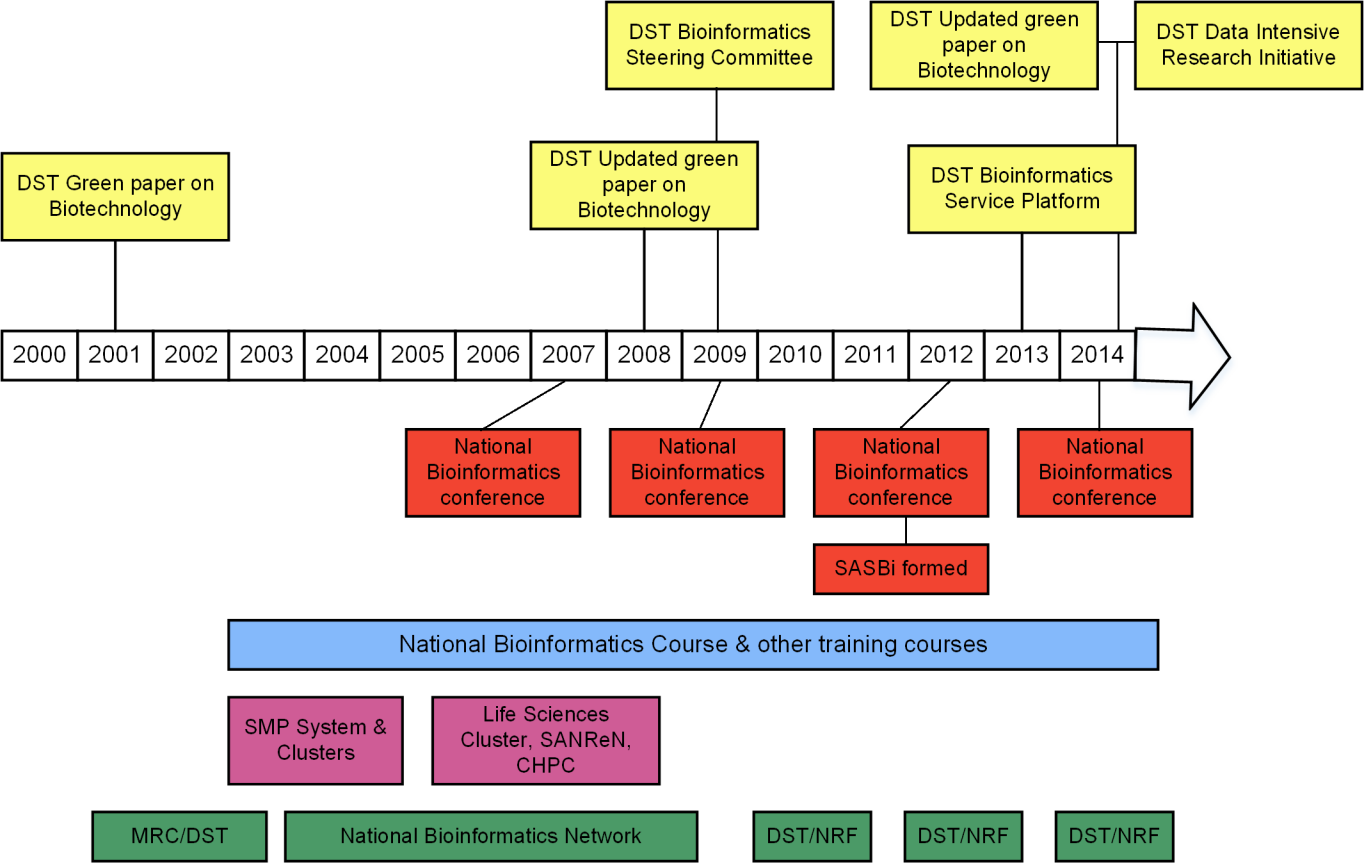
The policy, infrastructure, training, and funding landscape of computational biology in South Africa (2000–present). Government policy on computational biology was driven largely by the DST (above timeline arrow), while bioinformatics training courses have been a constant feature of this landscape since 2003. There was a major period of infrastructure investment from 2002–2007, from the NBN, but national funding has now become available only every two years. National bioinformatics conferences also occur in two-year intervals (details in text).

Following closure of the NBN initiative, the DST formed a steering committee for Bioinformatics in 2009, which facilitated bioinformatics research and fellowships disseminated through the NRF, commencing in 2010 and repeated every two years. Funding for a national Bioinformatics Service Platform (BSP) was granted in 2013, and the appointment of a coordinator is currently underway (discussed further below). Further support for bioinformatics research has been provided through the DST/NRF South African Research Chairs Initiative (SARChI), and three chairs are currently supported in the broad bioinformatics area (Bioinformatics and Public Health Genomics, Bioinformatics of African Populations, Mechanistic Modelling of Health and Epidemiology). Fig 1 provides a summary timeline of the major events impacting the development of bioinformatics in South Africa.

### International influences on the development of bioinformatics in South Africa

Several international initiatives influenced the development of bioinformatics in South Africa. In the early 1990s, the European Molecular Biology Network (http://www.embnet.org/) established the first bioinformatics network in Africa, with nodes similar to EMBNet Nodes in Europe. The first African Node was set up at SANBI, followed by many others (http://www.embnet.org/about/nodes). The World Health Organization’s TDR also provided funding to support establishment of an African Regional Training Centre for Bioinformatics and Applied Genomics in South Africa. As part of this activity, many training workshops were presented across Africa. Another approach to set up a large network within Africa, with leadership in South Africa, was led by the Centre National de la Recherche Scientifique (CNRS) in collaboration with NEPAD. This network funded the start of the ASBCB (African Society for Bioinformatics and Computational Biology) Bioinformatics conferences (ASBCB later joined forces with the International Society for Bioinformatics and Computational Biology [ISCB] to run these conferences) and workshops and mapped the bioinformatics structure in Africa (http://www2.lirmm.fr/france_afrique/20060708_NEPAD.pdf). These international activities helped to establish the initial bioinformatics network structure in South Africa, which in turn attracted national government recognition and commitment to advancing bioinformatics in the country.

More recently, international initiatives are having a profound influence on South African bioinformatics. The NIH-funded H3ABioNet Pan African bioinformatics network (www.h3abionet.org) is part of the Human Heredity and Health in Africa (H3Africa: www.h3africa.org, discussed later) initiative [10] and is coordinated from the University of Cape Town (UCT) with six additional nodes in South Africa. This project is building bioinformatics capacity on the continent and, as a result, is boosting bioinformatics activities in South Africa. The award of funding for H3ABioNet in South Africa prompted the DST to commit funds for bioinformatics support personnel at the Centre for High Performance Computing (CHPC) in Cape Town and to continue funding national bioinformatics courses for postgraduates.

## Infrastructure Development for Bioinformatics

Originally, costs for bandwidth in South Africa were prohibitive and bandwidth was low, with SANBI having a relatively good connection initially at a dedicated 1 Mbps in 2003. The lack of bandwidth posed huge challenges to the development of bioinformatics, and especially to accessing large international databases. The situation slowly improved in a disjointed fashion in different institutions, but progressed substantially only from 2006, with the establishment by the DST of the South African National Research Network (SANReN) and TENET (The Tertiary Education and Research Network of South Africa; an Internet Service Provider run by the universities). SANReN built a national fiber backbone with speeds up to 10 Gbps. TENET operated the national network and procured international bandwidth [11]. Currently, TENET has a 10 Gbps link to Amsterdam on the SEACOM (submarine cable operator) cable and a 10 Gbps link on the WACS (West Africa Cable System) cable to London, and manages transit to international networks including Géant (http://www.tenet.ac.za). This has led to substantial improvements in bandwidth availability for reduced costs. Although network bandwidth remains limited compared with many other countries and the high latency caused by physical distance imposes challenges, bandwidth has now reached the point where it is feasible to transfer terabyte-size datasets internationally.

The first major infrastructure for bioinformatics computing was Silicon Graphics SMP systems installed at SANBI in 1997, followed by the installation of Cray SV1 in 2002. Subsequently, the NBN funded the installation of some high-end SMP systems and clusters in the period from 2003–2009. Many of the active bioinformatics groups run their own clusters, and carry the bulk of the workload of bioinformatics in South Africa. However, national facilities have been a very valuable resource for the community. An Intel-funded cluster for Life Sciences was set up at the Council for Scientific and Industrial Research in 2005 for use by the greater bioinformatics community. The CHPC was established in 2007 with the installation of an IBM cluster with 160 cores and two IBM p690 systems. Later acquisitions include IBM BlueGene P in 2008 and a Sun M9000 SMP system with 2 TB of RAM in 2009. The current cluster is a heterogeneous cluster of Oracle and Dell machines with a peak performance of 61 teraflops and over 7,000 cores and 480 TB of external storage [11]. A significant upgrade is currently planned with some dedicated resources for bioinformatics. A new DST-funded Data Intensive Research Initiative of South Africa is being established to support large-scale data storage. There have also been a number of examples of the use of public cloud facilities.

In examining the successes and shortcomings to date, there are two key lessons: (1) lack of specialized skills (bioinformaticists, bioinformatics data analysts, systems administrators, and specialist programmers) has been more of a stumbling block than access to hardware resources; and (2) the most expensive equipment does not necessarily make the biggest contribution, even when the upfront capital costs are very low (e.g., through a donation). Thus, initiatives such as the CHPC appointing two dedicated specialized bioinformatics staff members to support the bioinformatics community are particularly positive.

## Bioinformatics Education and Training

In South Africa, bioinformatics teaching is mainly offered at the postgraduate level, though some universities offer short undergraduate modules. Students accepted to postgraduate bioinformatics degree programs have a variety of backgrounds, including biological sciences, computer science, mathematics, physics, and statistics. Although there are only a handful of universities giving formal qualifications in bioinformatics in the country, a number of other universities have projects in which bioinformatics is either the full focus or a component of the research (See Table 1 for programs available and students trained). Students from these universities are supported by attending national bioinformatics courses or training activities, by attending bioinformatics modules at their own university, or through collaboration with bioinformatics groups.

**Table 1:** Bioinformatics degree programs offered at some of the South African universities. The numbers shown are for registrations or graduations where the content is bioinformatics. The key research areas of each institution are included.

University	Start of group	Area of research	Graduates to date
Nelson Mandela Metropolitan University (NMMU)	2014	Structural bioinformatics of immune receptors	
Rhodes University (RU), Research Unit in Bioinformatics (RUBi) http://rubi.ru.ac.za/	2003*	Structural Bioinformatics, Comparative Genomics, Tool Development, Database Development	2 Honors, 25 MSc (1 yr)**, 3 MSc, 2 PhD
Stellenbosch University (US) Molecular Systems Biology group	2000	Computational Systems Biology; mechanistic modelling of health and epidemiology; yeast, bacterial and plant systems biology; model database; model simulation software.	17 MSc, 9 PhD
UCT, Computational Biology Group http://www.cbio.uct.ac.za/	2003	Bioinformatics & Systems Biology of infectious diseases, population genetics and disease, development of new algorithms and visualization for high-throughput biology	17 Honors, 16 MSc, 9 PhD
University of Free State http://cbio.ufs.ac.za/	2005	Epigenomics	4 MSc
University of KwaZulu Natal (UKZN) http://lifesciences.ukzn.ac.za/Staff/Biotechnology/biotech_staff/Biotech_pmb/pillayc.aspx; http://www.bioafrica.net	2009	Redox Systems Biology, HIV and tuberculosis (TB) drug resistance, evolutionary biology, development of online databases and bioinformatics tools	8 MSc, 5 PhD
University of Pretoria (UP), Bioinformatics and Computational Biology Unit http://www.up.ac.za/centre-for-bioinformatics-and-computational-biology	2002	Cancer Genomics, Bacterial Genomics	32 Honors, 29 MSc, 10 PhD
UWC, SANBI http://www.sanbi.ac.za	1996	Genome assembly, identification of human disease genes using NGS, transcriptomics, network biology, viral genomics and phylogenetics, pathogen genomics and drug resistance, evolutionary biology, in-silico drug lead discovery, semantic database development.	26 MSc, 29 PhD
University of Witwatersrand (Wits) http://www.bioinf.wits.ac.za	2003	Novel algorithm development, genome-wide association study (GWAS) and population structure, NGS analysis for studying gene/disease interaction	2 Honors, 6 MSc, 2 PhD

*Activities suspended 2005–2009

**Machanick and Tastan Bishop 2015 [12]

### National bioinformatics courses

Bioinformatics was not traditionally part of undergraduate curricula in South Africa, so students registering for postgraduate degrees in bioinformatics tend to start with little formal training in the field. In response, the NBN developed joint courses compulsory for NBN-funded students that introduced them to a range of bioinformatics topics and developed their programming and other technical skills. The first courses were run over two semesters of eight weeks each, but the length and curriculum has been refined over time to account for changing needs, costs, and realistic length of time students can spend away from their research projects. Today, these courses run for seven weeks at the start of each academic year and are aimed at students registered for dissertation-only postgraduate bioinformatics degrees at South African universities. At the start, we relied heavily on international trainers, but now all trainers are based within the country, a testament to the building of local training and skills capacity. In addition to exposure to a range of topics, computer science and mathematics students learn basic molecular biology, while biology-trained students learn programming. All the students benefit from networking opportunities and sharing skills and experience with their peers. These courses, which are supported financially by the DST, are extremely popular and oversubscribed, so we are considering running two such courses in different geographical locations where training is needed, and in the future. The substantial number of applications from bench scientists to attend these courses highlights the current gap in the training of bioinformatics users.

### Other bioinformatics training initiatives

In addition to these national courses for training bioinformaticists, different universities and the NBN have run short specialized training courses funded by their own institutions, foreign funding agencies, the NBN or directly by the DST. Some have hosted short courses organized through collaborative grants that individual researchers have with other countries or through funds raised from initiatives such as the NRF Knowledge, Interchange, and Collaboration program. Many of these have focused on training biological scientists in the use of bioinformatics tools, but some have also been tailored towards extending the skills of bioinformaticists. Some examples are provided below but are by no means comprehensive.

Several researchers at South African universities have hosted Ensembl and other roadshows on specific databases or tools, including Galaxy (http://galaxyproject.org/). They have also organized training around specialized topics or techniques, such as microarray, proteomics, or NGS data analysis. These are initiated through personal contacts, and funding sources vary. While such short-course training is not coordinated in South Africa, in many cases funding has been shared, particularly where the course runs in multiple locations in the country. This provides a cost-effective means of training many people in each region. These courses have focused mainly on training researchers in South Africa, but several courses have been hosted in the country and targeted to those both internal and external to the country. For example, SANBI hosted annual WHO TDR workshops for African scientists, and the results of one of these workshops are included in a recent Science publication [13]. UKZN and Medical Research Council in Durban have also served as a hub of education and training on bioinformatics, training people both locally and abroad. International organizations that have had a training presence in the country are the ICGEB (International Centre for Genetic Engineering and Biotechnology), Wellcome Trust, EMBO (European Molecular Biology Organization), EMBNET, and the NIH, through H3ABioNet. Training workshops were also held in 2011 in Cape Town as part of the ISCB Africa ASBCB conference on bioinformatics [14]. In addition to providing training courses, several South African academics are actively involved in GOBLET (Global Organization for Bioinformatics Learning, Education and Training) [15].

The DST, in addition to supporting national bioinformatics courses, has also made provision for financial support of necessary skills development and training needs, in the form of workshops and conferences as required by the bioinformatics community. It extends its training initiative through international cooperation partnership agreements, e.g., EMBO and Cooperation in Science and Technology (COST) training, as well as a number of other DST-driven platforms and Centers of Competence to enhance the bioeconomy of South Africa [8]. Also, as part of the Bioinformatics and Functional Genomics (BFG) program, which is administered through the NRF to fund bioinformatics research, the DST supports the development of human capacity and the creation of BFG skills for bio-based innovation and technology initiatives. Through this, South Africa is creating a growing pool of postgraduate students equipped to support bioeconomy developments, solutions, and knowledge relevant to the South African biotechnology industry, and national initiatives such as the Technology Innovation Agency (TIA), Centers of Excellence, and Research Chairs. It also aims to promote collaboration between academic institutions, science councils, and industry in South Africa, thus linking bioinformatics education to other national initiatives. There are various other training mechanisms to complement as well as to strengthen the training needed to bridge the gap between the academia and industry. Examples include internships offered by the TIA, NRF, and H3ABioNet, train-the-trainer programs, and a Knowledge Transfer Program (KTP) by the Centre for Proteomic and Genomic Research (CPGR).

## Development of a Bioinformatics Support Structure for Research and Industry

Bioinformatics capacity is essential in order to harness genomics research capacity, due to the large datasets generated and the complex analyses they require [16]. In 2008, the South African DST launched a Ten-Year Innovation Plan, with a “Farmer to Pharma” component that aimed to strengthen the bioeconomy and grow biotechnology in the country (http://www.dst.gov.za/images/pdfs/The%20Ten-Year%20Plan%20for%20Science%20and%20Technology.pdf. An important goal of the DST in funding bioinformatics initiatives was to provide bioinformatics support to researchers in academia and industry, and many of the funded activities actively contributed to this mission. Support included providing advice on and help with bioinformatics analyses, as well as developing new tools and resources and skills required locally. One of the major focus areas in the bioeconomy strategy is health. Although there are many examples of the application of bioinformatics to agriculture and other focus areas in SA, here we only discuss how some of the specific bioinformatics needs in health-related fields are being addressed.

### Bioinformatics in health

Genomics, biotechnology, and specifically bioinformatics were areas that were identified as requisite for addressing the heavy burden of disease in South Africa, particularly diseases of poverty [17]. To date, medical genomics research in South Africa has focused almost exclusively on infectious diseases that include HIV, malaria and tuberculosis. Bioinformatics approaches are used to identify host, vector, and pathogen variation, and to identify variants and biomarkers of pathogenicity and multidrug resistance. Genomic approaches have also been applied to infectious disease control [18]. South Africa, however, is in the process of epidemiological transition from a major disease burden of infectious to noncommunicable diseases [19,20]. Furthermore, the advent of NGS technologies, their increasing accessibility, and decreasing costs have opened new avenues to understanding genotype–phenotype relationships in noncommunicable human disease [20], and the high genetic diversity of sub-Saharan African populations positions South Africa to conduct groundbreaking genomics research towards understanding genetic factors underlying human disease [21].

#### H3Africa initiative

In 2012, human health genomics research in Africa received a significant boost through the H3Africa program [10] jointly funded by the National Institutes of Health (US) and the Wellcome Trust (UK). Out of over 20 funded projects in this program, ten are led from South Africa. Bioinformatics capacity for health genomics research has specifically been prioritized within H3Africa through the H3Africa Bioinformatics Network (H3ABioNet) mentioned previously. H3ABioNet [22] is building capacity by developing computing infrastructure, bioinformatics graduates, and training programs for genomics researchers. It is addressing all computational aspects of genomics projects from providing support for patient databases, through full data analysis, to enabling submission to public repositories. This is being achieved through training and pooling of resources across the network partners to develop a solid support structure.

#### HIV drug resistance databases

A good translational example of the usage of bioinformatics applications for health in South Africa was the setup of HIV drug resistance databases. The scaling up of access to antiretroviral therapy (ART) in southern Africa had an extraordinary effect on extension of survival among those infected [23]. However, a major challenge is the development of drug resistance. Bioinformatics methods are widely used in developed countries to identify drug resistance mutations in HIV patients on ART [24]. As part of a collaborative project between the Wellcome Trust Africa Centre at UKZN and the MRC, the two leading drug resistance databases in the world, housed at Stanford University (Stanford, US) and the Rega Institute (Leuven, Belgium), were transferred to bioinformatics servers in South Africa and made publicly available [25,26]. These databases were published in HIV Drug Resistance regional guidelines [27] and are implemented in the national treatment programs of Botswana and South Africa [28].

#### South African Human Genome Program

The South African Human Genome Program (SAHGP) was launched in January 2011, with support from the DST. Its goals are to: (1) develop capacity for genomic research in southern Africa, (2) establish a sustainable resource for genomic research, and (3) translate the information and knowledge into improvements in human health [29]. Significant work has gone into the planning of the SAHGP, especially with regard to ethical, legal, and social issues, which are particularly important in our environment [30]. A pilot phase of the SAHGP is currently under way; 24 complete genomes have been sequenced and are currently under analysis. This provides an important training opportunity for enabling South African scientists to build capacity for large-scale sequence data analysis.

#### Computational systems biology for health

From its initiation, the NBN held a broad computational biology view of bioinformatics, and it included Computational Systems Biology as one of its course topics. Systems biology formed part of research groups in several of the network nodes. In addition to training students in the field, systems biology software tools e.g., a simulation engine, PySCeS [31], and a curated model database JWS Online [32] were developed and are publically available. The model database is linked to several international scientific journals and in large European systems biology projects as part of a data and model management structure. Many systems biology projects are applied in biotechnology or health-related projects; the recently started SARChI chair in “Mechanistic modelling of health and epidemiology” with a strong link to the South African Centre for Epidemiology and Mathematical Analysis in Stellenbosch is an excellent example. The JWS Online database is funded via large international Systems Biology grants for which it is used as part of the data and model management team (e.g., SysMO I and II, and currently ERASysApp). The database is maintained at Stellenbosch University (SUN) with mirror sites at the University of Manchester and the Vrije Universiteit in Amsterdam. Funding to maintain the databases has been guaranteed for at least ten years after the current funding round ends. The South African part of the initiative is funded via the long term SARCHI initiative.

### Establishment of a National Bioinformatics Support Platform

Improved affordability and availability of high-throughput technologies to the South African scientific community has generated increasingly complex genomic datasets, with a concomitant increase in the need for bioinformatics support. Since the dissolution of the NBN, the existing bioinformatics groups have generally been able to secure funding for research, but not for service provision. Despite this, there has been an expectation from peers at their universities that they should play a role in providing support to researchers. This puts them in a difficult situation as salaries for support staff need to be covered and they should derive some benefit from their involvement. The analysis, however, doesn’t always lead to publications. In addition, this may address some of the needs of researchers located near competent bioinformatics groups, but not those of other researchers or the biotechnology community. In response, a proposal for a National BSP was developed by a Steering Committee, and subsequently funded by the DST. The platform is currently being established and staffed, and will provide a bioinformatics support structure for researchers in the academic and commercial sectors throughout the country. The BSP will respond to service requests nationally based on expertise and managed by a central national helpdesk. Although centrally coordinated, BSP staff will be seconded to institutions who demonstrate significant and ongoing need for support. The BSP will also take over custodianship of national bioinformatics training and education that lies outside of university responsibilities. The hope is that the BSP will fill the current gap in bioinformatics service provision that has come to be expected by scientists.

## Local and International Links

The computational biology community in South Africa is continuing to promote local and international links through crossinstitutional grants from individuals, large networks, and more formal organizations or societies. Increasingly, grants are awarded to multicenter networks, for example, a BFG grant from the National Research Foundation of South Africa has been awarded to partners at UWC, UCT, UP, SUN, University of the Witwatersrand, University of Limpopo, and the Agricultural Research Council for the development of computational resources to support analysis of Southern African human genomes. Separately, SUN and UWC are funded under this program to investigate genomic and proteomic determinants of *Mycobacterium tuberculosis* phenotypic characteristics. Collaboration across Africa is also increasing, for example the Medical Research Council of South Africa funds a Flagship project to develop computational tools for tuberculosis research, with partners at UWC, UKZN, SUN, UCT, and the University of Benin. The South Africa–Kenya grant funds a program to characterize miRNA in disease vectors, with partners UWC and ICIPE in Kenya.

Outside Africa, there are many active collaborations, for example the Africa Centre for Health and Population Studies at UKZN collaborates with the UCT Computational Biology group and the Swiss Institute of Bioinformatics (SIB) on the Swiss–South Africa grants and with CNRS, Leuven Katholeic University, University of Manchester, and Erasmus MC H2020 EC project (VIROGENESIS: developing of next generation tools for virus NGS data). In another example, the Computational Biology Group at UCT partnered with the European Bioinformatics Institute (EBI) and eight other institutions in Europe and the US on a European Union (EU) FP7 project (contributing to the InterPro database), and has ties with several European bioinformatics groups, including SIB, facilitated by Swiss–South Africa and Spain–South Africa grants. Research from the group feeds directly into the Swiss-based TubercuList, one of the key reference databases for *Mycobacterium tuberculosis*.

### South African Society for Bioinformatics (SASBi)

Local bioinformatics groups were adversely affected by the closing of the NBN, but the impact went beyond the financial, since the NBN also provided a voice and a discussion platform for the bioinformatics community in the country. In order to bring the bioinformatics community together, the community recently established SASBi (http://sasbi.weebly.com/). The aim was to support newly established bioinformatics research groups, to enhance interactions among people related to bioinformatics, and to form a representative body to interact with government. An interim steering committee was formed to establish the national society, and this committee produced a draft constitution. The society was inaugurated on 11 September 2012 at a meeting during the first joint conference of the South African Genetic Society (SAGS) and the bioinformatics community. The second joint SAGS/SASBi conference took place in September 2014. Both DST and BSP have provided financial support which made it possible to support 71 students to attend the conference. SASBi has interacted with the DST on a number of occasions, e.g. it conducted a national survey for bioinformatics needs, and discussed the BSP proposal. During the second joint conference, a SASBi Student Society was formed, and has already been affiliated with the international society (ISCB).

### International societies and networks

Many members of SASBi are also members of international societies such as the ASBCB and the ISCB. At least four South African bioinformaticians have previously been or are currently members of the ISCB Board of Directors, and one has served two terms as President of ASBCB. South Africa hosted one of the ISCB Africa ASBCB Bioinformatics conferences in Cape Town in 2011. The SASBi student society was affiliated with ISCB in March 2015, and the affiliation of SASBi with ISCB will be initiated this year now that the society has been in existence for a minimum of two years. Individual bioinformatics researchers also have numerous international collaborators both on the continent and abroad, some of which are described above. A notable example of a large collaborative network is the previously mentioned H3ABioNet network, which links seven local bioinformatics groups with over thirty groups continent-wide and two in the US, and is led from South Africa. The project also links the bioinformatics community with genomics researchers and clinicians in Africa through the wider H3Africa consortium, which consists of over 350 members in 18 African countries.

The EMBNet has been a direct and indirect supporter of bioinformatics initiatives in South Africa. They have, for example, provided numerous reviewers for national grant applications, identified partners for various successful and unsuccessful grant applications, sponsored national conferences, and provided technical support for bioinformatics queries through their mailing lists. They have run training courses in the country and recently facilitated the link between South Africa and the EU COST action, SeqAhead (http://www.seqahead.eu/). South Africa was the first African country to join the Action BM1006 called the NGS network. SeqAhead offered various workshops on NGS data and aimed to galvanize the implementation of efficient workflows for NGS data storage, retrieval, sharing, and analysis.

## Conclusions

**Fig 2:**
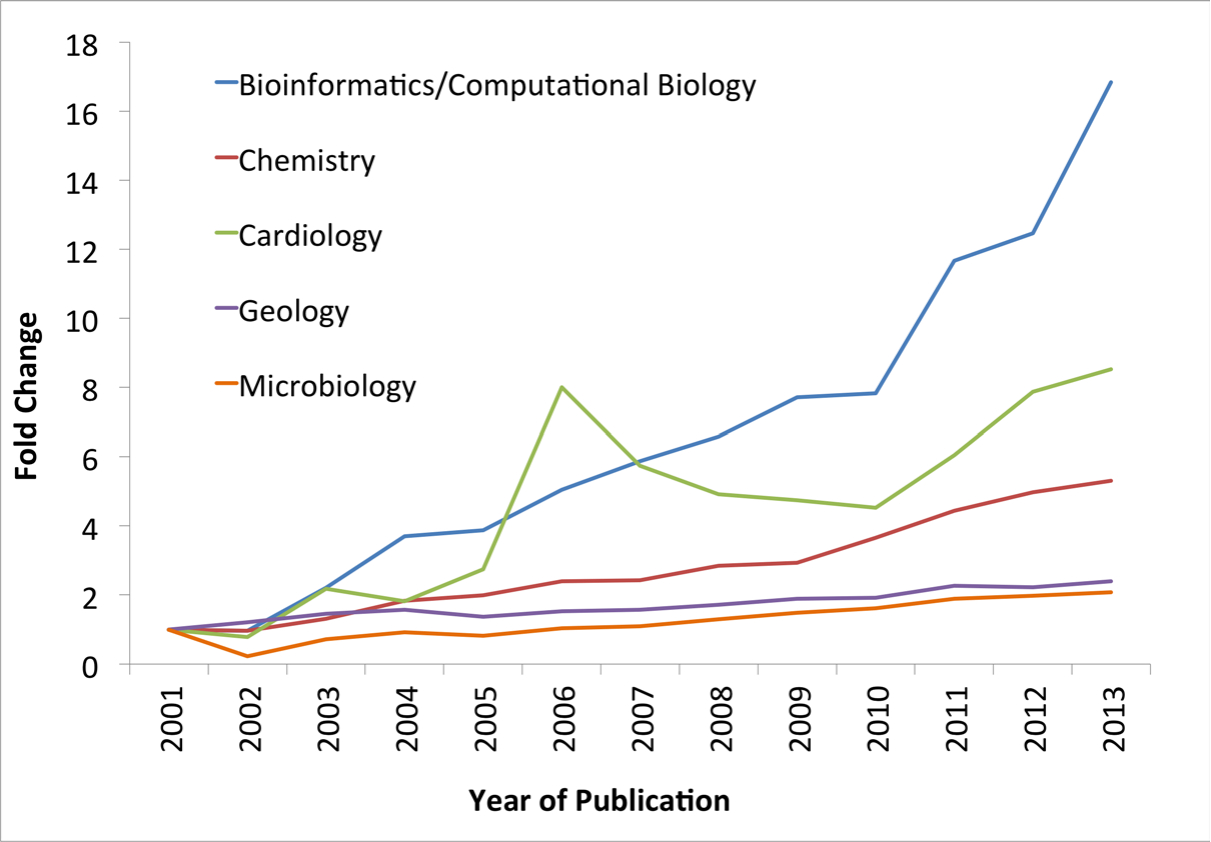
Relative increase in bioinformatics and computational biology publications from South Africa compared to publications in other subject areas. Fold change indicates relative increase in publication numbers compared to number of publications in 2001.

Bioinformatics in South Africa started with a single group at one South African university, but developed rapidly through the injection of funds from the DST via the NBN initiative. The NBN provided a springboard for the development of bioinformatics skills in postgraduate students and bench scientists, and these new initiatives are ensuring a continuation of this momentum. Since then, the Bio-Innovation Unit of the DST has invested in several key bioinformatics-based initiatives, including, the BFG program; the BSP; and the SAHGP. They have contributed to a significant increase in bioinformatics outputs including trained postgraduate students, research publications (see Fig 2), patents, and bioinformatics tools and resources. There have also been downstream impacts, such as creation of new bioinformatics jobs, small business startups, and enhanced bioinformatics service delivery. Examples of these include HealthQ Technologies (http://www.healthq.co), a start-up company originating at Stellenbosch with founding members from the computational systems biology group; and Electric Genetics, which developed StackPACK from EST (Expressed Sequence Tag) clustering and transcript reconstruction software originating from SANBI, which was subsequently used by Affymetrix as part of its expression array design process. Electric Genetics also cosponsored and hosted the first ever two-part Open Bio Hackathon with O’Reilly, which was a major driver towards the first BioPerl publication. To demonstrate the impact on publications, we conducted a search of PubMed (on 22 January 2015) using the following search parameters: [“subject area"] and "south africa"–"working in partnership with the European Bioinformatics Institute" and “.ac.za”, where “subject area” was [“bioinformatics” or “computational biology”], “chemistry,” “cardiology,” “geology,” “microbiology”. Patents and citations were excluded. We chose to simplify the search and use the broad terms bioinformatics and computational biology, as it is challenging to identify all bioinformatics publications by journal or topic alone. We therefore included the other subjects to demonstrate the relative differences in impact using broad subject terms. It is evident that the number of bioinformatics-related publications in South Africa has increased substantially since 2001, with a sharper rise than other subjects over the same time period.

Some of the key achievements of the South African bioinformatics community lie in training, research, and infrastructural capacity development. The universities have collectively trained 130 Masters and 60 PhD students in bioinformatics, many of which have gone on to academic positions or placements in prestigious institutes abroad, including Harvard and the EBI. Bioinformatics courses in South Africa are increasingly being taught by exclusively local trainers, many of whom are frequently invited to give training courses in Africa and abroad. Several groups have contributed to or developed resources used by the international scientific community, one example being the HIV drug resistance database. The strength and international recognition of the community is reflected in the participation of South African academics in key positions in international societies, such as ISCB and ASBCB, partnerships with international research groups in successful funding proposals, and the increase in publications in international bioinformatics journals. One of the highlights, though, is the NIH award for H3ABioNet, which is one of the largest single awards to an African institution.

Throughout the history of the development of bioinformatics in SA, many lessons have been learnt, some of which are described above. The take-home message is that commitment to bioinformatics capacity development by the government has been key to the success of many of the bioinformatics units in the country. The step-up provided by the NBN enabled groups in multiple universities to hire dedicated bioinformatics staff members in an environment where universities were not forthcoming in creating new positions. In some cases, the success of the groups convinced the institution of the importance of bioinformatics and the need to support it. The establishment of these bioinformatics units also facilitated the procurement of further funding from the NRF as well as international funding agencies, thus increasing outputs and enabling these units to become internationally competitive in the field.
